# Factors Related to the Length of Stay for Patients With Schizophrenia: A Retrospective Study

**DOI:** 10.3389/fpsyt.2021.818254

**Published:** 2022-01-24

**Authors:** Peng Cheng, Lirong Wang, Lizhi Xu, Ying Zhou, Li Zhang, Weihui Li

**Affiliations:** ^1^National Clinical Research Center for Mental Disorders and Department of Psychiatry, The Second Xiangya Hospital of Central South University, Changsha, China; ^2^Xiangya School of Medicine, Xiangya Hospital, Central South University, Changsha, China

**Keywords:** schizophrenia, the length of stay, retrospective study, public mental health (PMH), ROC (receiver operating characteristic) analysis

## Abstract

**Background:**

The length of stay (LOS) of patients with schizophrenia has been a wide concern of researchers. Reasonable management of the LOS to achieve a balance between quality of treatment and efficient medical source allocation has become a significant issue in clinical work in psychiatry. Figuring out the factors related to the LOS of schizophrenia patients can help optimize its management by the hospital.

**Method:**

The essential information of patients was obtained from the electronic medical record system. The variables were divided into the following kinds: demographic, clinical, and biochemical. Univariate analysis and multivariate analysis were conducted to find the potential factors related to the LOS of schizophrenia patients. Receiver operating characteristic analyses were conducted to evaluate the accuracy of judging the LOS of the regression model.

**Result:**

A total of 1,160 patients with schizophrenia were enrolled in our research. Our results demonstrated that the status of unmarried (single, separated, divorced, or widowed) and the abnormality of thyroid-stimulating hormone (TSH) were risk factors for the longer LOS of schizophrenia patients. The area under the curve was 0.576, which meant that the regression model had a certain predictive value.

**Conclusion:**

To our knowledge, this research is the first study to analyze the effect of various factors, including the biochemical index, on the LOS of a single type of mental disorder. Marital status and TSH were proven to be related to the LOS of schizophrenia patients. The results of this study provided reference factors of LOS for clinical psychiatry, which will be helpful to the management of hospitalization and in optimizing the allocation of medical sources.

## Introduction

Schizophrenia is a chronic, severe, and disabling disorder, which causes impairments in everyday functioning and involves major functional domains of independence in residence, productive activities, and social interactions. Many schizophrenia patients, accompanied with irritability caused by delusion and hallucination, were usually unmanageable by their families, unless they were admitted to the psychiatry department. This situation led to a serious problem in a way that many schizophrenia patients tended to have long-stay hospitalization or to be continually hospitalized. Moreover, schizophrenia patients were unable to work due to deterioration in social function. Both two factors lead to a heavier financial burden not only to themselves but also to the social medical system. Concerning the climbing prevalence of schizophrenia and the huge fundamental population in China (1), rational and efficient allocation of medical care sources to these populations was important (2). In recent years, length of stay (LOS) of psychiatric patients with schizophrenia has been a wide concern of researchers. Reasonable management of the LOS to achieve a balance between quality of treatment and efficient application of medical sources had become a significant issue in clinical work in psychiatry.

Since the “deinstitutionalization” movements for psychiatric inpatients during the 1970's and the positive expectation of atypical antipsychotics, the LOS of schizophrenia patients has gradually become shorter (3). While in many cases the LOS was reduced (especially in medically and financially strapped countries), this was more inspired by limiting the psychiatric care cost brought about by long psychiatric hospitalization of schizophrenia patients instead of the simple purpose for functional recovery in communities (4, 5). Based on a related survey, the LOS of schizophrenia patients in a developing country was less than that in a developed country, such that the mean LOS of patients with schizophrenia in China was 73.3 ± 42.2 days (6), while it was 78 ± 76 days in South Korea (7), 90–120 days in Israel (8), 96.6 days in Canada (9), and 290.6 days in Japan (10). Certainly, factors associated with the LOS of schizophrenia patients were not just found in the financial field. Many studies were conducted by researchers in different samples, and they demonstrated a series of various kinds of potentially influential factors on the LOS of schizophrenia patients. Most of them were demographic and clinical, which can be divided into the following categories: demographic variables (age/gender/marital status/employment/ethnicity) (11–14), behavioral variables (history of assault/aggressiveness/involuntary hospitalizations) (15, 16), and clinical variables (higher discharge dose of antipsychotics/the number of previous admissions) (13).

Although many factors have been confirmed to be associated with the LOS of patients with schizophrenia from various countries and regions, to our knowledge, analogous studies were rather limited in China. Especially after the introduction of China's new Mental Health Law in 2013 (17), the diagnosis and curation of mental disorders, including schizophrenia, were clearly defined. This law particularly emphasized that the admission and discharge of patients with schizophrenia should be based on the voluntary principle of the patient. Even if patients were confirmed with irritability, suicide ideation, or other uncontrollable dangerous situations that lack insight, the admission and discharge should still be under the supervision of the medical department of the government. Only patients diagnosed with severe psychiatric disorders as recognized by the Chinese Healthcare Commission and by a certified forensic psychiatry assessment, with an accompanying high risk or behavior of hurting themselves or other people, were eligible for compulsory admission to appointed hospitals sent by the company of said individuals or the local police station (17). Thus, the human rights of patients with psychiatric disorders were protected sufficiently by the new Mental Health Law. The new provisions of the Mental Health Law certainly had potential effects on the LOS of Chinese schizophrenia patients compared to the previous situation wherein related laws are lacking. China, as a developing country with tremendous medical pressure brought about by the biggest population size in the world, needs to analyze the possible factors related to the LOS of schizophrenia patients. Figuring out the factors related to the LOS of schizophrenia patients in the context of the new Mental Health Law in China can guide the management of a hospital, including but not limited to determining priorities, improving the medical quality, and allocating resources reasonably.

Additionally, most of the previous studies lacked the biochemical variables of schizophrenia patients, which have been demonstrated by accumulating evidence to be related to the development and prognosis of this disorder. We chose thyroid-stimulating hormone (TSH) and lipoprotein as our target biomedical characters to investigate in this study after filtering related researches. Previous basic psychiatric research have demonstrated that TSH was essential for the regulation of dopaminergic, serotonergic, glutamatergic, and GABAergic networks. The pathways of neurotransmitters mentioned above were correlated to the mechanism of schizophrenia (18). A systematic review analyzing the function of thyroid hormones, which enrolled 19 studies, demonstrated that the level of TSH might be abnormal in either first-episode schizophrenia patients or multiple-episode schizophrenia patients. Moreover, the level of TSH was fluctuating along with the course of schizophrenia (19).

As for lipoproteins, despite the wide application of atypical antipsychotics for its better therapeutic effects and fewer adverse events than typical antipsychotics (20), the side effect of metabolic disturbances caused by atypical antipsychotics, including dyslipidemia, cannot be ignored. Clinical observation illustrated that those patients using atypical antipsychotics would have weight gain and lipid and glucose abnormalities in just 2 to 3 months, and such changes continue over time (21, 22). These changes can increase the comorbidity risk of cardiovascular disease, which will undoubtedly have a negative effect on the prognosis of schizophrenia patients (23). A comparative study demonstrated that schizophrenia patients had higher levels of serum LDL, but lower levels of HDL, than health controls (24). The result mentioned above hinted that the level of lipoproteins had an effect on the tolerance to antipsychotic treatments of schizophrenia patients and then consequently affected the LOS. Thus, based on previous studies, we hypothesized that TSH and lipoproteins had potential effects on the LOS of schizophrenia patients.

As recent data concerning the LOS of schizophrenia patients in China is missing, the purpose of the current study was to examine various kinds of variables (demographic/clinical/biochemical) within a large inpatient sample in China, to determine the factors related to the LOS of schizophrenia patients, and then to provide a theoretical reference for the future management of psychiatry departments and the allocation of medical resources.

## Materials and Methods

### Participants and Study Design

This research was an epidemiological retrospective study, obtaining data from the Second Xiangya Hospital of Central South University, which was one of the four mental health centers in China. The essential information of patients was derived from the electronic medical record system. As for the criteria of diagnosis, only patients who were individually diagnosed with schizophrenia without other mental disorders were eligible to enroll in our sample group. In our research, all the patients with schizophrenia were admitted voluntarily based on clause 30 of the China Mental Health Law, concerning the admission of psychiatric patients, and relevant regulations of the Second Xiangya Hospital of Central South University. The inclusive time of admissions of patients in our study ranged from 2012 to 2020. We divided all the independent variables into the following categories: demographic variables (i.e., gender, age, job status, marital status, and ethnicity), clinical variables (i.e., previous admission and evaluation of current hospitalization), and biochemical variables (i.e., TSH and lipoprotein cholesterol). The dependent variable was the LOS of schizophrenia patients, which was coded as a binary variable by setting “short LOS” as ≤28 days and “long LOS” as > 28 days.

The reasons why “28 days” was chosen to be the criterion of long LOS were based on both patients and hospitals. From the perspective of patients, many local organizations or companies considered 28 days as the maximum of short-term leave, which had no obvious adverse effect on their salary. More than 28 days of LOS, which meant a long-term leave, would dramatically reduce their income. From the perspective of hospitals, as the Chinese medical insurance system had a strict limitation on the cost of hospitalization, 28 days was a common range of LOS limitation applied by many hospitals to restrict the LOS so as to meet relevant rules promulgated by the medical insurance department. Additionally, some previous studies about the LOS conducted in psychiatry also chose 28 days as the cutoff of the long-LOS group for analyzing factors related to the LOS, which means that this time range was recognized in these kinds of study with different backgrounds. In general, 28 days was a reasonable criterion of the long LOS, with both clinical and practical significance in the current research. Coding the LOS as a dichotomous variable is due to the skew distribution observation in the original data of the LOS. Meanwhile, the dichotomous nominal variable with odds ratio has more reference value than the linear outcome variable to clinical work. This methodological measurement has become accepted in other research analyses (14, 25). All the variables in our samples have been automatically filtered to ensure that there was not any missing data. The variables in our study are listed in Table 1.

**Table 1:** Univariate analysis of variables related to length of stay (LOS).

**Variable categories**	***N*** **(%)**	**Short LOS**	**Long LOS**	***t***/**χ^2^**	***P-*** **value**
**Demographic variables**					
**Gender**					
Male	550 (47.4%)	395 (71.8%)	155 (28.2%)	0.186	0.666
Female	610 (52.6%)	445 (73.0%)	165 (27.0%)		
Age (M ± SD)	25.36 ± 10.03	25.7 ± 10.22	24.47 ± 9.48	1.882	0.06
**Job status**					
Unemployed	791 (68.2%)	566 (71.6%)	225 (28.4%)	0.918	0.338
Employed	369 (31.8%)	274 (74.3%)	95 (25.7%)		
**Marital status**					
Single, separated, divorced, or widowed	926 (79.8%)	643 (69.4%)	283 (30.6%)	20.343	<0.001
Married	234 (20.2%)	197 (84.2%)	37 (15.8%)		
**Ethnicity**					
Ethnic Han	1,143 (98.5%)	829 (72.5%)	314 (27.5%)		0.584
Ethnic minority	17 (1.5%)	11 (64.7%)	6 (35.3%)		
**Clinical variables**					
**Number of previous admissions**					
0	928 (80.0%)	680 (73.3%)	248 (26.7%)	1.726	0.189
≥1	232 (20.0%)	160 (69.0%)	72 (31.0%)		
**Evaluation of hospitalization**					
Unimproved	19 (1.6%)	17 (89.5%)	2 (10.5%)	2.814	0.093
Improved	1,141 (98.4%)	823 (72.1%)	318 (27.9%)		
**Biochemical variables**					
**Thyroid-stimulating hormone**					
Abnormal	130 (11.2%)	83 (63.8%)	47 (36.2%)	5.38	0.02
Normal	1,030 (88.8%)	757 (73.5%)	273 (26.5%)		
**Lipoprotein cholesterol**					
**High-density lipoprotein cholesterol**					
Abnormal	341 (29.4%)	251 (73.6%)	90 (26.4%)	0.344	0.557
Normal	819 (70.6%)	589 (71.9%)	230 (28.1%)		
**Low-density lipoprotein cholesterol**					
Abnormal	188 (16.2%)	142 (75.5%)	46 (24.5%)	1.092	0.296
Normal	972 (83.8%)	698 (71.8%)	274 (28.2%)		

### Measures

#### Demographic Variables

These variables were obtained at the admission of each patient, including age (coded as ratio data), gender (female or male), job status (unemployed or employed), marital status (unmarried situations and married), and ethnicity (ethnic Han and ethnic minority).

#### Clinical Variables

These variables were obtained pertaining to occasions when the patients got treatment in the hospital, including the number of previous admissions (0 or ≥ 1) and evaluation of hospitalization (unimproved or improved). The “evaluation of hospitalization” was defined based on the Clinical Global Impression—Improvement (CGI—I) scale. The CGI—I is a widely used measurement for the global assessment of patients with psychiatric illnesses, quantifying the overall impression of a clinician of the clinical change of the psychiatric conditions of patients (26, 27). The questionnaire of each schizophrenia patient was finished by their corresponding resident doctor. Only the scores of CGI—I ranging from 1 to 3 (respectively corresponding to very much improved, much improved, and minimally improved) were coded as “improved” in the current research.

#### Biochemical Variables

These bio-variables were also obtained at the admission of each patient similar to the demographic variables. Both TSH and lipoprotein cholesterol (HDL and LDL) levels were measured based on the blood sample obtained at the beginning of hospitalization. All the biochemical indexes were set as dichotomous variables, with the reference being the normal range of healthy people.

### Statistical Analysis

Both univariate analyses and multivariate analyses were conducted in the current research to investigate the potential factors related to the LOS of schizophrenia patients. Chi-square analysis and ANOVA analysis were performed to establish whether differences existed across the LOS concerning demographic, clinical, and biochemical variables. Factors were preliminarily considered to be associated with LOS at *P* ≤ 0.10. Moreover, we considered all the variables mentioned above for which a significant effect may be assumed to the LOS of schizophrenia patients (*P* ≤ 0.1 in the univariate analysis). A multivariate regression model was built to identify the extent of prediction of variables in the current study to the LOS of schizophrenia patients. Statistical significance was considered significant at two-tailed *P* < 0.05. Then, receiver operating characteristic (ROC) analyses were conducted to evaluate the accuracy of judging the LOS of schizophrenia patients of this regression model. The data was analyzed with SPSS, version 25.0 (IBM Corp., New York, USA).

## Results

### Description Statistics and Univariate Analysis

Information of a total of 1,970 patients was obtained from the electronic medical record system. After filtering out patients with any missing value, a total of 1,160 patients with schizophrenia were enrolled in our research. The admission of all the patients in our research was based on voluntary principle as per both the requirements of the China Mental Health Law and the rules of the Second Xiangya Hospital of Central South University. The details of our sample are shown in Tables 1, 2. From the perspective of demography, the ratio of men to women was roughly equal (male = 47.4% and female = 52.6%); the age of the sample was relatively young, and they were mainly unemployed and unmarried. People of ethnic Han comprised most of the sample (ethnic Han = 98.5%). The result of the clinical variables indicated that one-fifth of our sample have a previous experience of admission of psychiatry. The quality of hospitalization measured by CGI showed that the conditions of most of the schizophrenia patients improved (unimproved = 1.6% and improved = 98.4%). HDL has the highest abnormal proportion among the biochemical indexes (29.4%), which was followed by LDL (16.2%). The abnormal proportion of TSH was 11.2%, which was the lowest among the biochemical variables (see the details in Table 1).

**Table 2:** Description of the length of stay of patients with schizophrenia.

**Category**	***N*** **(%)**
Length of stay, LOS [M (SD)]	23.38 ± 11.72
Short LOS	840 (72.4%)
Long LOS	320 (27.6%)

As for the LOS, the mean LOS of patients in the research was 23.38 ± 11.72 days. The proportion of the short LOS was 72.4%, while that of the long LOS was 27.6%. The details are shown in Table 2. Univariate analysis was conducted between demographic variables, and the dichotomous LOS variable demonstrated that the short-LOS group was statistically more likely to be older, married, with unimproved hospitalization, and had a normal level of TSH. Conversely, the long LOS group was more likely to be younger, unmarried (single, separated, divorced, or widowed), with improved hospitalization, and had an abnormal level of TSH (see Table 1).

### Multivariate Analysis

A multivariate logistic regression model was built to examine the role of demographic, clinical, and biochemical factors, which were tested with statistical significance in the univariate analysis, in association with the LOS of schizophrenia patients. The results are shown in Table 3.

**Table 3:** Multivariable logistic regression analysis.

**Variables**	**OR**	**95%CI**	***P-*** **value**
Age	1.005	0.990–1.022	0.506
**Marriage status**			
Single, separated, divorced, or widowed	1 [reference]	NA	<0.001
Married	0.393	0.253–0.611	
**Evaluation of hospitalization**			
Unimproved	1 [reference]	NA	0.107
Improved	3.393	0.769–14.967	
**Thyroid-stimulating hormone**			
Abnormal	1 [reference]	NA	0.018
Normal	0.623	0.422–0.921	

**Figure 1:**
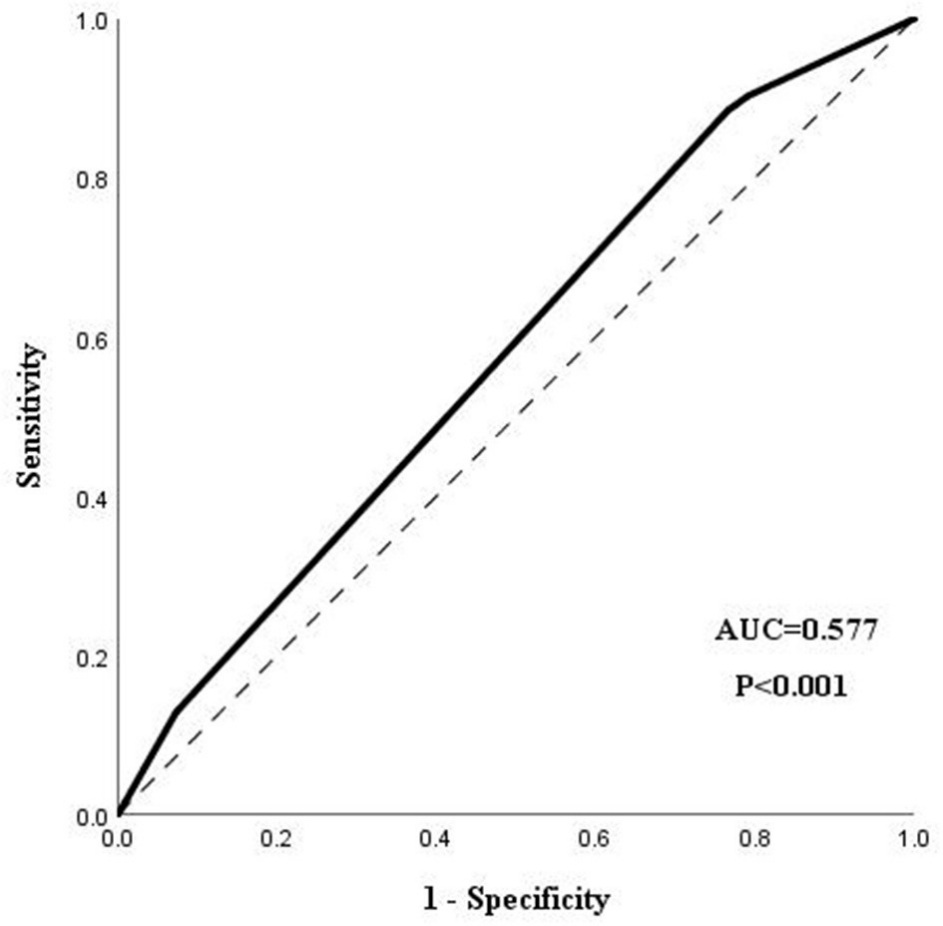
The ROC analysis of the regression model.

Marital status and TSH were significantly associated with a longer LOS in our regression model. Married patients with schizophrenia were more likely to have a shorter LOS than patients without this emotional relationship (OR = 0.393, 95%CI = 0.253–0.611). Patients with a normal level of TSH were commonly discharged earlier than patients with an abnormal level of TSH (OR = 0.623, 95%CI = 0.422–0.921). Age and the evaluation of hospitalization were not statistical factors related to the LOS of schizophrenia patients. The ROC analysis demonstrated that the area under the curve (AUC) was 0.577 (95%CI = 0.541–0.613), with a certain significance to predict the LOS of schizophrenia patients. The result of the ROC analysis is shown in Figure 1.

## Discussion

This retrospective cohort study analyzed the potential factors, including demographic, clinical, and biochemical indexes, related to the LOS of patients with a monotonous diagnosis of schizophrenia. Our results demonstrated that married status and a normal level of TSH were independently associated with a shorter LOS of schizophrenia patients. In other words, the status of unmarried (single, separated, divorced, or widowed) patients and the abnormality of TSH were risk factors for a longer LOS in schizophrenia patients.

In this research, the LOS of patients with schizophrenia was lower than in a previous similar study about LOS (6). The reasons which caused the difference of LOS between these two studies with totally different backgrounds were diversified, including, but not limited to, the epidemiology variance brought by time, geography, and sampling variability and the different medical insurance limitations set by local governments. Further multi-center analysis needs to be conducted to obtain a more universal conclusion.

Marital status was defined as a dichotomous variable—married and unmarried (including single, separated, divorced, or widowed)—in this study, which meant that, for most cases, the group of unmarried patients tended to have less support (especially in the spiritual context). A previous study illustrated that the resilience of married patients with schizophrenia was better than those who were unmarried (28), which was owing to the comfort and support brought about by a stable married relationship. Some follow-up studies (29, 30) suggested that resilience acted as a significant role in the recovery process of schizophrenia patients. Additionally, recent researches have shown that resilience may have a potential influence on the treatment outcome of schizophrenia (31, 32), even acting as a protective factor of improved recovery (33). Marital status, as a demographic factor that can reflect resilience, was related to the recovery process of schizophrenia patients, which was proven by the results of this study. As a non-intervening retrospective design study, the result of our study demonstrated that a stable married relationship could promote the recovery of schizophrenia in the real world, therefore decreasing the LOS of schizophrenia patients.

Our study results indicated that schizophrenia patients with a dysfunction of TSH had a longer LOS than patients with normal function. TSH has been widely recognized to be related to schizophrenia. Previous studies showed that patients with hyperthyroidism tend to have more risks of psychotic symptoms; conversely, hypothyroidism patients were more likely to have negative symptoms of schizophrenia (34, 35). A schizophrenia patient with an unnormal level of TSH was at a much higher risk of not only thyroid disorder but also psychiatric symptoms than normal patients. Additionally, in consideration of the fact that many antipsychotics had an adverse effect on the thyroid (36, 37), the condition of patients with initial TSH abnormality might get worse due to the adverse effect of antipsychotics, aggravating the psychotic symptoms or negative symptom and thus prolonging the LOS of schizophrenia patients and even leading to other clinical problems. Our study results indicated that schizophrenia patients with a dysfunction of TSH had a longer LOS than patients with normal function.

Besides this, lipoprotein cholesterols, even though a factor without statistical significance in our results, need to be mentioned, although previous studies illustrated that lipoprotein cholesterol was related to the prognosis of schizophrenia. The adverse change caused by the change of LDL or HDL was chronically pathological (e.g., weight gain caused by endocrine disorder and then increase in the risk of cardiovascular diseases), usually without affecting the single course of antipsychotic treatments. Thus, as this retrospective study only enrolled information of a single admission, the single LOS of schizophrenia patients had no significant relationship with the lipoprotein cholesterol. However, lipoprotein cholesterol was perhaps related to the LOS of schizophrenia patients in the long run. Follow-up studies need to be conducted in the future to figure out the long-term relationship between lipoprotein cholesterols and the LOS of schizophrenia patients.

## Limitation

Although potential factors with clinical reference significance related to the LOS of schizophrenia patients were found in our study, some limitations still need to be mentioned. Firstly, our research lacked data on the psychological measurements of psychotic and associated symptoms, an important index reflecting the severity of schizophrenia, which was maybe related to the LOS. Secondly, even this retrospective research only enrolled a single mental disorder. The variance of anti-psychotic treatments in the hospitalization may still have a non-negligible effect on the prognosis of patients, which would affect the LOS of schizophrenia patients. As the purpose of this study was to try to analyze the independent effect of different variables on the LOS at admission during hospitalization, further research need to be conducted on the premise of grouping the participants based on the treatments, thus figuring out the effect of antipsychotic treatments on the LOS of schizophrenia patients. Thirdly, as our research did not involve situations of compulsory admission, in view of the different effects between compulsory admission and voluntary admission, more studies need to be conducted to figure out the factors associated with the LOS in the sample of compulsory admissions. Fourthly, the AUC of the model in this research was 0.577, which was close to a random model. This result might be caused by the inadequate variables enrolled in our study, such as education levels, reminding us to enroll more variables potentially related to the target objective during the process of data collection in future work.

## Conclusion

To our knowledge, this research is the first study to analyze the effect of various factors, including the biochemical index, on the LOS of a single type of mental disorder. Marital status and TSH were proved to be potential factors related to the LOS of schizophrenia patients by a regression model. The results of this study provided reference factors of LOS for clinical psychiatry, which will be helpful in the management of hospitalization and optimizing the allocation of medical sources.

## Data Availability Statement

The raw data supporting the conclusions of this article will be made available by the authors, without undue reservation.

## Author Contributions

PC and LW: data collection, literature review, and manuscript drafting. YZ, LX, and LZ: managed the ethical review process. WL: manuscript drafting and revision. All authors read and approved the final manuscript.

## Funding

This study was supported by the Natural Science Foundation of Hunan Province, China (No. 2020JJ5844 to LZ), Natural Science Foundation of Hunan Province, China (No. 2018JJ2592 to WL), Hunan Key Research and Development Program (No. 2018SK2136 to WL), and Natural Science Foundation of China (82171518 to LZ).

## Conflict of Interest

The authors declare that the research was conducted in the absence of any commercial or financial relationships that could be construed as a potential conflict of interest.

## Publisher's Note

All claims expressed in this article are solely those of the authors and do not necessarily represent those of their affiliated organizations, or those of the publisher, the editors and the reviewers. Any product that may be evaluated in this article, or claim that may be made by its manufacturer, is not guaranteed or endorsed by the publisher.
